# A molecularly imprinted electrochemical sensor integrated with conversion factor T strategy for rapid and selective determination of lutein esters in marigold extracts

**DOI:** 10.48130/els-0026-0010

**Published:** 2026-08-24

**Authors:** Ziyu Zhu, Benshu Chen, Aiqin Luo, Yong Cong, Wei Li, Liquan Sun, Axin Liang

**Affiliations:** 1 Key Laboratory of Molecular Medicine and Biotherapy, the Ministry of Industry and Information Technology, School of Life Science, Beijing Institute of Technology, Beijing 100081, China; 2 Lacare Nutritional Technology (Beijing) Co., Ltd, Beijing 100192, China

**Keywords:** Electrochemical sensor, Ni_3_(HITP)_2_-MOF, Molecularly imprinting, Lutein esters, Conversion factor T

## Abstract

Establishing a comprehensive analytical method capable of accurately reflecting the composition of major lutein esters and enabling their rapid quantification is essential for the quality evaluation of marigold products. In this study, the major lutein esters present in marigolds were first identified by HPLC-MS, and a novel 'conversion factor T' strategy was established. Using lutein dipalmitate as the reference marker, the contents of other major lutein esters were calculated based on their relative conversion factors. To further simplify the analytical procedure, a molecularly imprinted electrochemical sensor based on conductive Ni_3_(HITP)_2_-MOF was developed for the rapid determination of lutein dipalmitate. The introduction of Ni_3_(HITP)_2_-MOF enhanced the electrode conductivity by approximately fourfold compared with the bare electrode. The sensor exhibited a wide linear range of 0.01–100 μM and a low detection limit of 8.20 nM toward lutein dipalmitate. Four major lutein esters, namely lutein dimyristate, lutein myristate-palmitate, lutein dipalmitate, and lutein palmitate-stearate, were quantified, and the corresponding conversion factors (T*_d_*, T*_m_*, and T*_p_*) were determined as 0.903, 0.964, and 1.035, respectively. The proposed strategy integrates rapid electrochemical sensing with conversion factor analysis, enabling indirect quantification of multiple lutein esters from a single target analyte. Furthermore, the method was successfully applied to the quality evaluation of marigold extracts and commercial eye care products, demonstrating its potential for rapid routine analysis and quality control.

## Introduction

Commercial lutein is predominantly sourced from marigolds. Initially, lutein, serving as a colorant, was extensively applied in poultry feed and the food industry^[1]^. Subsequently, epidemiological evidence demonstrating the health benefits associated with the consumption of carotenoids has further propelled the market for lutein dietary supplements and marigold-based extracts. Currently, these products are also widely utilized for the fortification of foods and beverages^[2]^. Marigold aligns well with the 'food as medicine' concept and thus enjoys broad applications^[3]^.

Supplements rich in lutein have been linked to a reduced risk of developing age-related macular degeneration (AMD). Oxidative stress generates reactive oxygen species (ROS), including free radicals, hydrogen peroxide, and singlet oxygen^[4,5]^. These ROS can cause cellular damage, accelerate the aging process of the retina, and ultimately contribute to the progression of AMD^[6,7]^. Macular xanthophylls, including lutein and zeaxanthin, are incorporated into retinal membranes, where they contribute to retinal protection by scavenging ROS and reducing oxidative damage associated with lipid peroxidation and photooxidation^[8]^. In addition, Lutein induced the expression of Nrf2-target genes (antioxidant enzymes) and reduced the levels of inflammatory mediators to protect against inflammation-related neurodegenerative disorders^[9,10]^.

A large number of studies have been reported on lutein and lutein esters. High-performance liquid chromatography-mass spectrometry (HPLC-MS) is employed for the qualitative and quantitative analysis of lutein^[11,12]^. Lutein is acylated with saturated fatty acids in marigold petals and is mainly found in acidic fractions such as stearic acid (C18:0), palmitic acid (C16:0), myristic acid (C14:0), and lauric acid (C12:0)^[13]^. At present, the quantification of lutein from marigold extract, requires saponification to be carried out first to make lutein esters into free lutein^[14,15]^. Notably, the saponification method contributes to enhancing the convenience of detection^[16]^. Meanwhile, it avoids problems such as high-priced lutein ester standards and a lack of market sources^[17,18]^. However, the saponification process is cumbersome and requires heating, while lutein is unstable under heating conditions due to its structure^[19,20]^. Studies have suggested that saponification can result in the loss of carotenoid content in plant extracts^[21]^. Therefore, it is necessary to develop a more accurate and convenient quantitative analysis method.

Recently, electrochemical sensors have attracted increasing attention in food analysis and natural product quality evaluation because of their rapid response, high sensitivity, low cost, and simple operation^[22]^. Among them, molecularly imprinted polymer (MIP)-based electrochemical sensors exhibit excellent selectivity toward target analytes through the formation of specific recognition cavities. However, most reported electrochemical sensors are designed for the determination of a single compound and cannot directly provide comprehensive compositional information for complex natural products. Since lutein dipalmitate is the predominant lutein ester in marigold extracts, it can potentially serve as a representative marker for rapid analysis. Therefore, combining highly selective electrochemical sensing of lutein dipalmitate with a reliable multi-component conversion strategy may provide a practical approach for rapid and comprehensive evaluation of lutein esters.

As the 'gold standard' of quantitative analysis, the external standard method has become a common method for quantifying various substances^[23,24]^. Compared with external calibration alone, the application of internal standards can improve quantitative accuracy, as the analyte and reference compound undergo the same sample preparation procedures and chromatographic analysis conditions, thereby reducing potential variations during measurement^[25−29]^. In recent years, quantitative analysis of multiple components by single (QAMS) has become a widely used method to quantify the active ingredient contents in plant extracts^[30,31]^. By utilizing inexpensive, readily available, and stable reference compounds, QAMS allows for the simultaneous quantification of multiple similar compounds^[32,33]^. This increasingly adopted and effective method has significantly reduced the cost and complexity of experiments^[34]^.

In this study, the major lutein esters in marigold extracts were first identified and quantified by HPLC-MS. Based on the stable proportional relationship among the major lutein esters, a conversion factor (T) strategy was established using lutein dipalmitate as the reference marker. Furthermore, a molecularly imprinted electrochemical sensor based on conductive Ni_3_(HITP)_2_-MOF was constructed for the rapid and selective determination of lutein dipalmitate (Scheme 1). Unlike conventional analytical strategies that require individual standards for the quantification of each lutein ester or involve complicated saponification procedures, the proposed strategy integrates the high selectivity and rapid response of electrochemical sensing with the quantitative correlation provided by the conversion factor approach. Consequently, multiple major lutein esters can be simultaneously evaluated by determining only one representative analyte, significantly reducing the dependence on expensive reference standards and improving analytical efficiency. This integrated sensing-conversion strategy provides a convenient and reliable approach for rapid quality evaluation of marigold extracts and lutein-related products.

**Figure 1:**
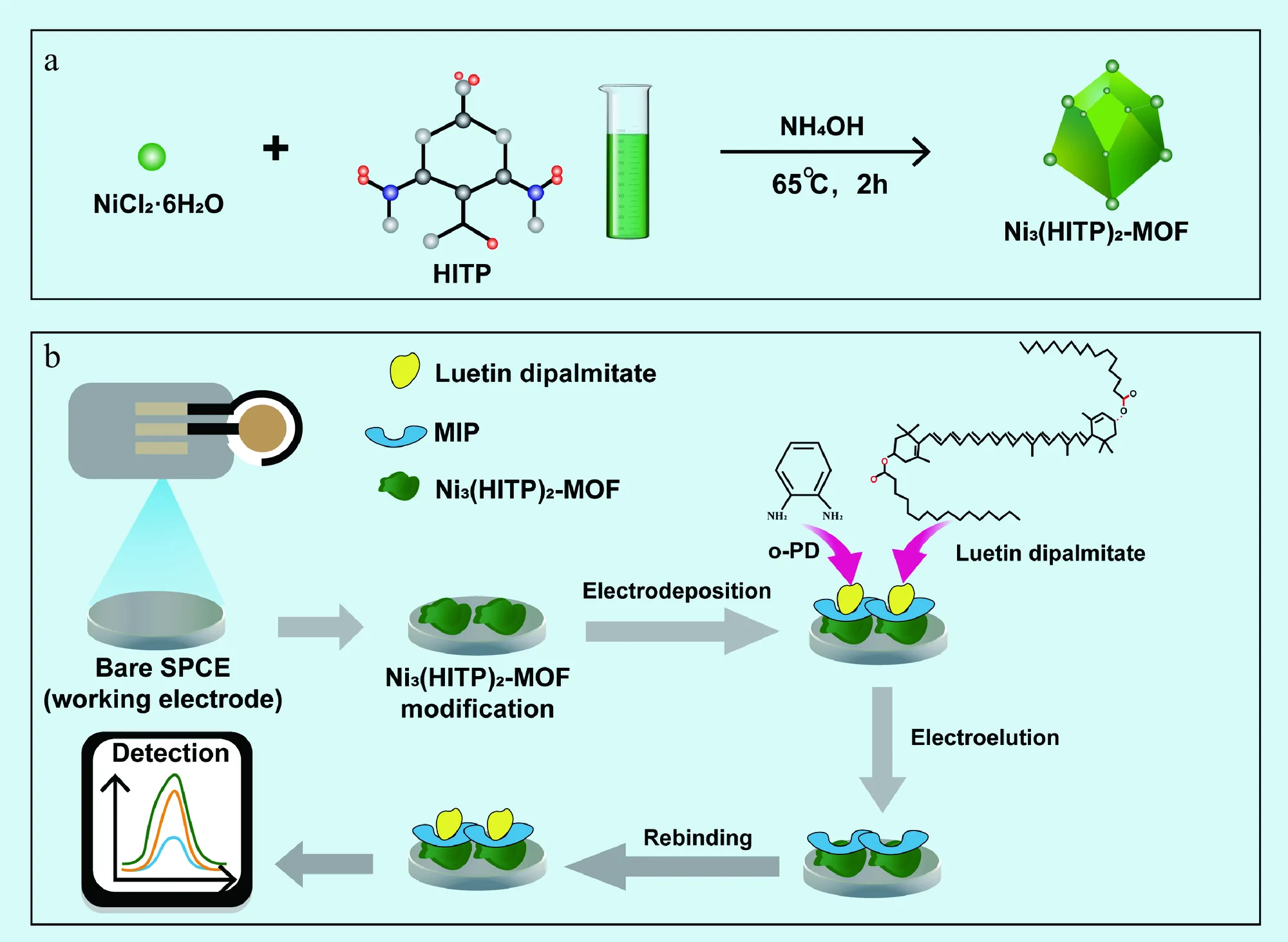
Fabrication procedure and sensing mechanism of the Ni₃(HITP)₂-MOF-based molecularly imprinted electrochemical sensor for selective lutein dipalmitate detection: (a) preparation procedure of Ni₃(HITP)₂-MOF; (b) fabrication procedure of the molecularly imprinted electrochemical sensor for selective lutein dipalmitate detection.

## Materials and methods

### Materials

HATP.6HCL (C18H24C16N6) was obtained from Shanghai Pedder Medical Technology Co., Ltd (Shanghai, China). Nickel dichloride hexahydrate (NiCl_2_·6H_2_O) was provided by Anhui Zesheng Technology Co., Ltd (Anhui, China). Acetic acid (HAc), potassium ferricyanide (K_3_Fe(CN)_6_), sodium chloride (NaCl), and potassium chloride (KCl) were supplied by Beijing Tongguang Fine Chemical Co., Ltd (Beijing, China). Sodium dihydrogen phosphate (NaH_2_PO_4_) and sodium hydrogen phosphate (Na_2_HPO_4_) were provided by Beijing Nuotron Chemical Technology Co., Ltd (Beijing, China). Acetone, creatinine, uric acid, ascorbic acid, and lactic acid were purchased from Nanjing Chemical Reagent Co., Ltd (Jiangsu, China). O-phenylenediamine (o-PD) was acquired from Beijing Warwick Chemical Co., Ltd (Beijing, China). Screen-printed carbon electrodes were purchased from Shenzhen Haoyang Technology Co., Ltd (Shenzhen, China). High performance liquid chromatography-mass spectrometry (HPLC-MS) purchased from Thermo Fisher (Beijing) Technology Co., Ltd rotary evaporator from Titan scientific lab (Shanghai, China), JHBE-20A flash extractor from Changyi Biological Co., Ltd (Henan, China); grinder and ultracentrifuge from Henan Jangyi Biological Co., ultrasonic and microwave cleaner from Beijing Tianlin Hengtai Co., Welchrom XBC18 chromatographic column (5 μm, 4.6 mm × 250 mm) was purchased from Yuexu (Shanghai) Technology Co., Ltd, ChromCoreC30 (3 μm, 4.6 mm × 150 mm) from NanoSpectrum Analytical Co., Ltd, the high performance liquid chromatography (HPLC) was purchased from Beijing Qinfang Science and Technology Company, Panna (Changzhou) Instruments Co., Ltd and the recirculating vacuum pump was purchased from Zhengzhou Great Wall Technology Co.

AR grade ethyl acetate, acetone, anhydrous ethanol, n-hexane were purchased from Beijing Tongguang Fine Chemical Company, HPLC grade ethyl acetate was purchased from Shanghai Myriad, HPLC grade acetonitrile, methanol was purchased from Shanghai Myriad, marigold, the control lutein dipalmitate was purchased from Shanghai Roen, and lutein dimyristate, Lutein myristic acid-palmitate, lutein dipalmitate, lutein myristate-palmitate, lutein palmitate-stearic acid ester were manufactured in the laboratory. Blueberry Lutein Ester care products were purchased from Beijing Lacare Company.

### Synthesis of Ni_3_(HITP)_2_-MOF

Dissolve 13.2 mg (0.028 mmol) of nickel chloride hexahydrate (NiCl_2_·6H_2_O) and 20 mg (0.019 mmol) of HATP·6HCl in 20 mL of ultrapure water and 0.6 mL of 14 mol/L ammonia solution. The resulting mixture is then placed in an open beaker and stirred at 65 °C for 3 h, producing a black powder. After centrifugation and filtration, wash the products with water for 48 h (12 h for each of the four washing steps). Finally, reflux the solid in acetone for 3 h, then vacuum-dry at 150 °C to obtain a black solid powder.

### Fabrication of the MIP/Ni_3_(HITP)_2_-MOF/SPCE sensor

A suspension of Ni_3_(HITP)_2_-MOF (1 mg/mL) was drop-cast onto the surface of the screen-printed carbon electrode (SPCE) working electrode and allowed to dry at room temperature, yielding the Ni_3_(HITP)_2_-MOF/SPCE electrode. Subsequently, the modified electrode was immersed in 10 mM phosphate-buffered saline (PBS, pH 7.4) containing 15 mM o-phenylenediamine (o-PD) and lutein dipalmitate as the template molecule. Electropolymerization was carried out by cyclic voltammetry within the potential range of −0.2 to 1.2 V for 12 cycles at a scan rate of 50 mV/s, forming a molecularly imprinted polymer film on the electrode surface. After polymerization, the electrode was thoroughly rinsed with ultrapure water to remove physically adsorbed species. The template molecule was subsequently removed by immersing the electrode in ethanol, generating specific recognition cavities complementary to lutein dipalmitate. The resulting molecularly imprinted sensor was denoted as MIP/Ni_3_(HITP)_2_-MOF/SPCE. For comparison, the non-imprinted sensor (NIP/Ni_3_(HITP)_2_-MOF/SPCE) was prepared under identical conditions in the absence of lutein dipalmitate

### Sample preparation

Samples of lutein, lutein dimyristate, lutein myristate-palmitate, lutein dipalmitate, and lutein palmitate-stearate were dissolved in the mobile phase, and the concentration of the sample was adjusted. The marigold powder was extracted by flash extraction with a soid-to-liquid ratio of 1:12. Then the mixture was filtered through a 0.22 μm filter membrane. The marigold extract and eye care products were saponified by adding 0.4 mol/L KOH/methanol and heating in a 55 °C water bath for 60 min.

### HPLC-MS analysis

The HPLC system is composed of SS 316L pumps, a PUD0010 Variable Wavelength Detector, and a Spak Autosampler. A reversed-phase Welchrom XBC18 (5 μm, 4.6 mm × 250 mm) Column Compartment was used for separation. The mobile phase was ethyl acetate, acetonitrile, and methanol (49:21:30, v/v/v). The analysis was carried out under isocratic conditions using a flow rate of 0.4 mL/min at room temperature (30 °C). The absorption wavelength was 445 nm. The APCI ion source was used in the positive ion scan mode; the scanning range was m/z 300–1,200, the positive ion discharge ion current was 4 μA, the ion transfer tube temperature was set at 350 °C, and the nebuliser temperature was 400 °C.

### Establishment of 'conversion factor T' method

Using lutein dipalmitate as the internal reference, the ratio of the peak area of the amount of other lutein esters per unit substance in the proposed marigold extract to that of lutein dipalmitate per unit substance was referred to as the 'conversion factor T', which was calculated by the formula below. Precision injection of different volumes of sample solution, HPLC detection and recording of chromatograms. According to the 'conversion factor T' formula, the conversion factors T*_d_* (T dimeric, T*_d_*), T*_m_* (T myristate, T*_m_*), and T*_p _*(T palmitate, T*_p_*) of lutein dipalmitate into lutein dimeric ester, lutein myristate-palmitate, and lutein palmitate-stearic acid ester were calculated.

1 * T= (Asi/ni)/(As/n) 
*

2 * n= m/M 
*

From Eqs (1) and (2):

3 $ T= (\boldsymbol{A}\boldsymbol{s}\boldsymbol{i}\times \boldsymbol{m}\times \boldsymbol{M}\boldsymbol{i})/(\boldsymbol{A}\boldsymbol{s}\times \boldsymbol{m}\boldsymbol{i}\times \boldsymbol{M}) $

The formula for content calculation is obtained from Eq. (3):

4 $ \boldsymbol{m}\boldsymbol{i}=(\boldsymbol{m}\times \boldsymbol{A}\boldsymbol{s}\boldsymbol{i}\times \boldsymbol{M}\boldsymbol{i})/(\boldsymbol{T}\times \boldsymbol{A}\boldsymbol{s}\times \boldsymbol{M}) $

where, *ni* is the amount of other lutein ester substances, mol; *n* is the amount of lutein dipalmitate substances, mol; *Asi* is the peak area of other lutein esters; *As* is the peak area of lutein dipalmitate; *m* is the mass of lutein dipalmitate, g; *mi* is the mass of other lutein esters, mg; *M* is the relative molecular mass of lutein dipalmitate; *Mi* is the relative molecular mass of other lutein esters.

### Instrument system verification validation

The impact of various flow rates, chromatographic columns, and column temperatures on the RSD (relative standard deviation, RSD) and RCF (Response Correction Factors, RCF) was examined to appraise the stability and durability of the 'conversion factor T method. Two columns, namely, Bonnasll AQ C18 and Welchrom XB C18, were adopted for analyses on a Qinfang LC-3020 HPLC system and a Panna Brave HPLC system, respectively. Moreover, the influence of column temperature (25, 30, 35, and 40 °C) and flow rate (0.1, 0.2, 0.3, and 0.4 mL/min) were assessed.

## Results

### Characterization of Ni_3_(HITP)_2_-MOF

The morphology and structure of the synthesized Ni_3_(HITP)_2_-MOF were characterized by SEM, XRD, and XPS. As shown in Fig. 1a, b, the Ni_3_(HITP)_2_-MOF exhibited an aggregated porous structure composed of interconnected nanoparticles. At a magnification of 20,000× (Fig. 1a), the material displayed irregular granular clusters with abundant interparticle voids, which could provide a large accessible surface area for molecular imprinting and analyte recognition. When the magnification was increased to 50,000× (Fig. 1b), the surface morphology became clearer, revealing a rough and highly porous architecture formed by densely packed nanostructures. Such a porous conductive framework is beneficial for mass transport and electron transfer during electrochemical sensing.

**Figure 1:**
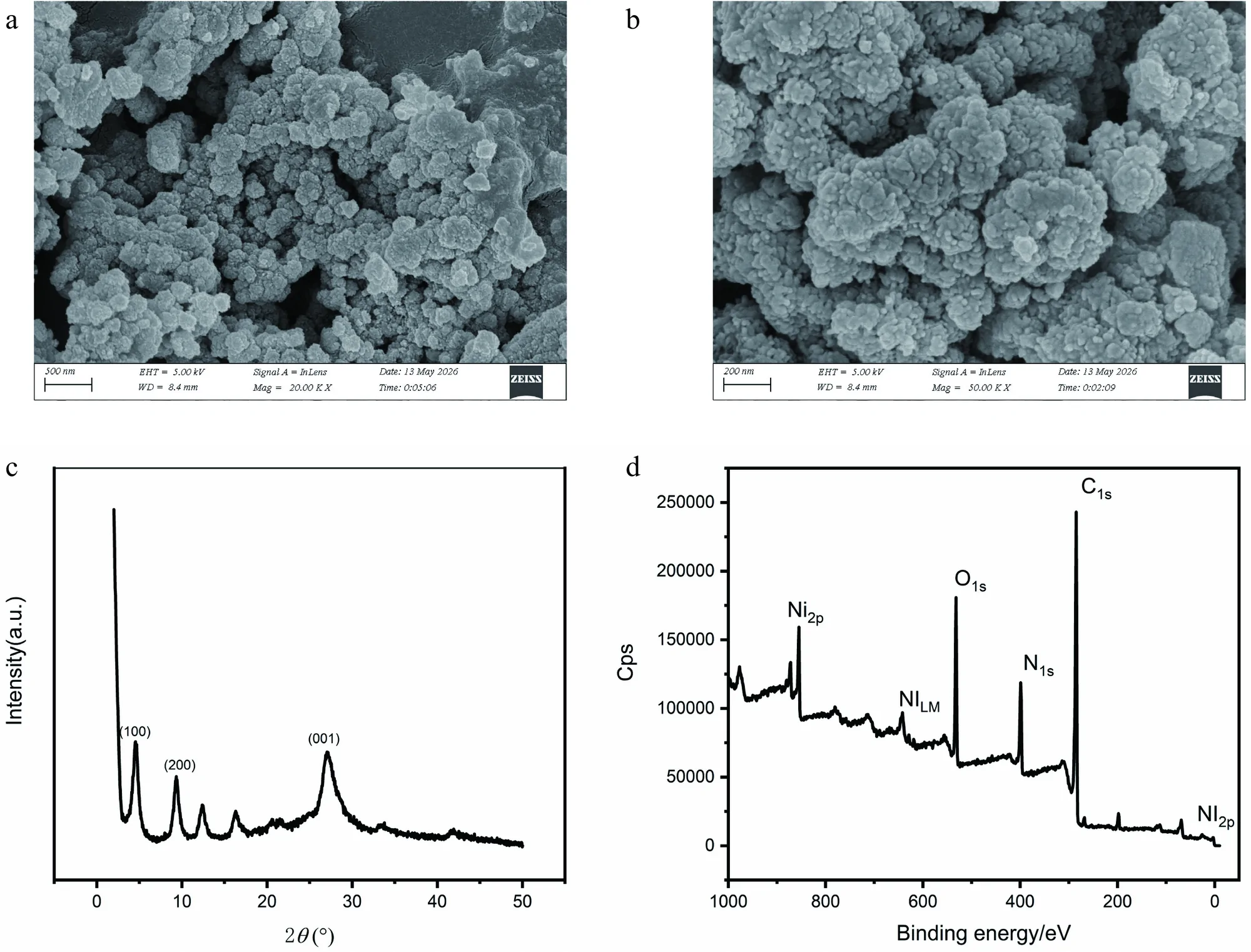
Characterization of Ni_3_(HITP)_2_-MOF. (a) SEM image of Ni_3_(HITP)_2_-MOF at a magnification of 20,000×. (b) SEM image of Ni_3_(HITP)_2_-MOF at a magnification of 50,000×. (c) XRD pattern of Ni_3_(HITP)_2_-MOF. (d) XPS survey spectrum of Ni_3_(HITP)_2_-MOF.

The crystal structure of Ni_3_(HITP)_2_-MOF was further investigated by X-ray diffraction (XRD). As shown in Fig. 1c, several characteristic diffraction peaks were observed, including the reflections corresponding to the (100), (200), and (001) crystal planes. These diffraction peaks are consistent with the reported crystalline structure of Ni_3_(HITP)_2_-MOF, confirming the successful formation of the conductive metal-organic framework.

The elemental composition of Ni_3_(HITP)_2_-MOF was analyzed by X-ray photoelectron spectroscopy (XPS). As shown in Fig. 1d, the survey spectrum revealed the presence of Ni, C, N, and O elements. The characteristic peaks of Ni 2p, C 1s, N 1s, and O 1s confirmed the successful incorporation of nickel ions and HITP ligands into the framework structure. The coexistence of these elements further verified the successful synthesis of Ni_3_(HITP)_2_-MOF.

The SEM, XRD, and XPS results collectively demonstrated the successful preparation of Ni_3_(HITP)_2_-MOF with a porous conductive structure, providing a suitable platform for the subsequent fabrication of the molecularly imprinted electrochemical sensor.

### Fabrication, optimization, and analytical performance of the MIP/Ni_3_(HITP)_2_-MOF/SPCE sensor

**Figure 2:**
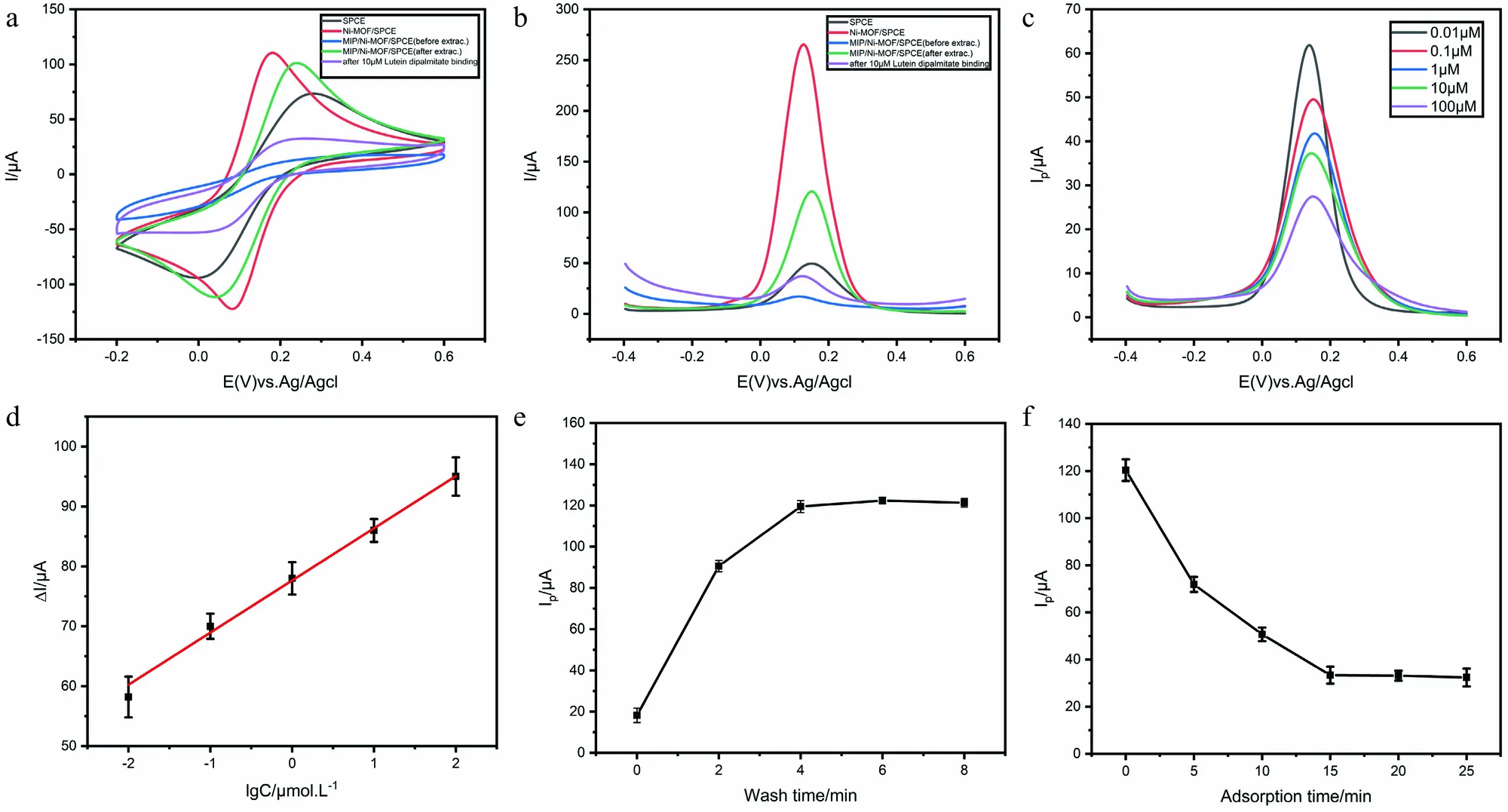
Fabrication, optimization and analytical performance of the MIP/Ni_3_(HITP)_2_-MOF/SPCE sensor. (a) Cyclic voltammetry (CV) responses of SPCE, Ni_3_(HITP)_2_-MOF/SPCE, MIP/Ni_3_(HITP)_2_-MOF/SPCE before template removal, MIP/Ni_3_(HITP)_2_-MOF/SPCE after template removal, and the sensor after rebinding with 10 μM lutein dipalmitate. (b) DPV responses corresponding to different stages of sensor fabrication and target rebinding. (c) DPV responses of the MIP/Ni_3_(HITP)_2_-MOF/SPCE sensor toward different concentrations of lutein dipalmitate (0.01–100 μM). (d) Linear relationship between the current change (ΔI) and the logarithm of lutein dipalmitate concentration. (e) Optimization of template removal time. (f) Optimization of adsorption (rebinding) time. The results demonstrate the successful construction of the MIP/Ni_3_(HITP)_2_-MOF/SPCE sensor, with enhanced recognition capability toward lutein dipalmitate and favorable analytical performance after optimization.

The electrochemical behavior of the fabricated MIP/Ni_3_(HITP)_2_-MOF/SPCE sensor was investigated by cyclic voltammetry (CV) and differential pulse voltammetry (DPV) . As shown in Fig. 2a, the bare SPCE exhibited a typical redox response. After modification with Ni_3_(HITP)_2_-MOF, the redox peak current increased significantly, indicating that the highly conductive MOF effectively facilitated electron transfer. Compared with the bare electrode, the peak current of the Ni_3_(HITP)_2_-MOF/SPCE increased by approximately fourfold, demonstrating the excellent conductivity of the synthesized material.

Following electropolymerization of o-phenylenediamine (o-PD) in the presence of lutein dipalmitate, the peak current decreased markedly due to the formation of a dense molecularly imprinted polymer film on the electrode surface, which hindered the diffusion of the redox probe. After template removal with ethanol, the peak current increased again, suggesting the successful formation of imprinted cavities. Upon rebinding with lutein dipalmitate, the current decreased once more because the template molecules reoccupied the recognition sites and partially blocked electron transfer. These results confirmed the successful fabrication of the molecularly imprinted sensor.

The corresponding DPV responses are shown in Fig. 2b. Similar trends were observed during electrode fabrication. The current response of Ni_3_(HITP)_2_-MOF/SPCE was substantially higher than that of the bare SPCE, whereas the MIP film significantly suppressed the electrochemical signal. After template removal, the current recovered due to the formation of accessible recognition cavities, while rebinding of lutein dipalmitate resulted in a pronounced signal decrease. The observed 'gating effect' further verified the successful preparation of the MIP/Ni_3_(HITP)_2_-MOF/SPCE sensor.

**Figure 3:**
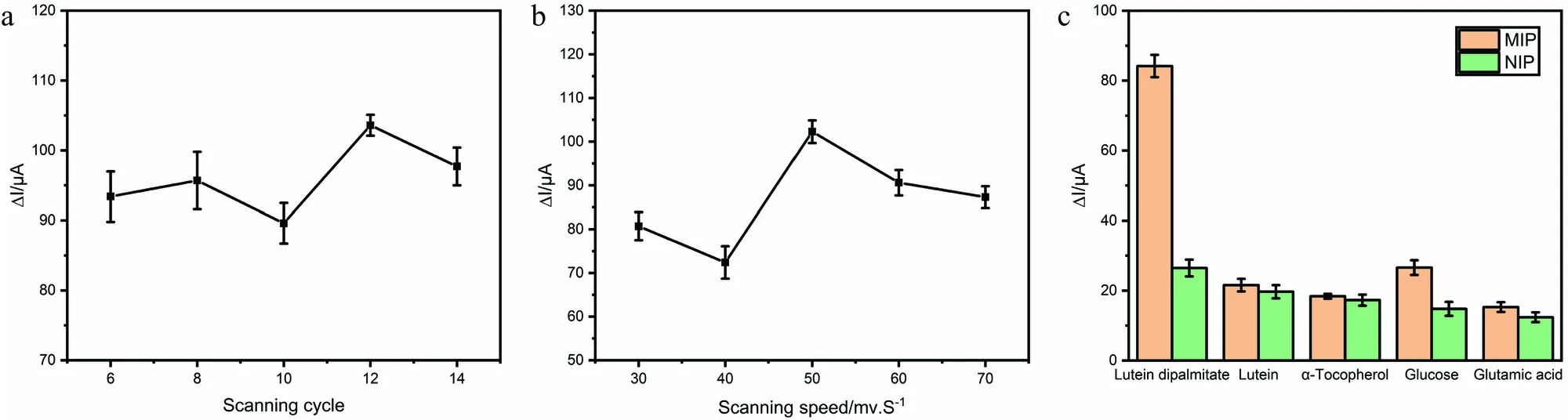
Optimization and selectivity evaluation of the MIP/Ni_3_(HITP)_2_-MOF/SPCE sensor. (a) Optimization of electropolymerization cycles for fabrication of the MIP/Ni_3_(HITP)_2_-MOF/SPCE sensor. (b) Optimization of scan rate during electropolymerization of the MIP/Ni_3_(HITP)_2_-MOF/SPCE sensor. (c) Selectivity evaluation of the MIP/Ni_3_(HITP)_2_-MOF/SPCE sensor. Current responses of MIP and NIP sensors toward lutein dipalmitate and potential interfering compounds, including lutein, *α*-tocopherol, glucose, and glutamic acid, at the same concentration. The MIP sensor exhibited a significantly higher response toward lutein dipalmitate than toward the interfering substances, demonstrating the successful formation of specific molecular recognition sites. Error bars represent the standard deviation of three independent measurements (*n* = 3).

The electropolymerization conditions were optimized by varying the number of CV cycles and scan rate. As shown in Fig. 3a, the current response increased with increasing polymerization cycles and reached a maximum at 12 cycles. Further increasing the cycle number resulted in a slight decrease in signal, likely due to excessive film thickness hindering mass transfer. Therefore, 12 cycles were selected as the optimal electropolymerization condition. In addition, different scan rates were investigated (Fig. 3b). The maximum sensor response was obtained at 50 mV/s, which was subsequently adopted for sensor fabrication.

The effects of template removal and rebinding time were also evaluated. As shown in Fig. 2e, the DPV response increased with increasing elution time and reached a plateau after 4 min, indicating complete removal of the template molecules. Therefore, 4 min was selected as the optimal elution time. The adsorption kinetics of lutein dipalmitate are shown in Fig. 2f. The current response gradually decreased with increasing incubation time and became stable after 15 min, suggesting that adsorption equilibrium had been achieved. Thus, 15 min was chosen as the optimal rebinding time.

Under the optimized experimental conditions, the sensor exhibited concentration-dependent DPV responses toward lutein dipalmitate over the concentration range of 0.01–100 μM (Fig. 2c). As the concentration of lutein dipalmitate increased, the peak current gradually decreased due to the occupation of imprinted cavities by target molecules. A good linear relationship was obtained between the current change (ΔI) and the logarithm of lutein dipalmitate concentration (Fig. 2d), covering three orders of magnitude. The sensor achieved a low detection limit of 8.20 nM, demonstrating high sensitivity for lutein dipalmitate determination. The selectivity of the MIP/Ni_3_(HITP)_2_-MOF/SPCE sensor was further investigated using several potentially interfering compounds, including lutein, *α*-tocopherol, glucose, and glutamic acid. As shown in Fig. 3c, the MIP sensor exhibited a significantly higher response toward lutein dipalmitate than toward the other tested compounds. Although lutein possesses a chemical structure similar to that of lutein dipalmitate, its response was substantially lower than that of the target analyte. In contrast, glucose, glutamic acid, and *α*-tocopherol generated only weak responses, indicating limited nonspecific interactions with the imprinted cavities. Furthermore, the corresponding NIP sensor displayed relatively low responses toward all tested compounds, including lutein dipalmitate. The significantly enhanced response of the MIP sensor toward lutein dipalmitate compared with the NIP sensor demonstrates the successful formation of specific recognition sites during the molecular imprinting process. These results confirm that the MIP/Ni_3_(HITP)_2_-MOF/SPCE sensor possesses excellent selectivity and molecular recognition capability for lutein dipalmitate.

The excellent conductivity of Ni_3_(HITP)_2_-MOF, together with the specific recognition capability of the molecularly imprinted polymer, enabled the sensor to provide rapid, sensitive, and selective detection of lutein dipalmitate, making it suitable for subsequent quantitative analysis of lutein esters in marigold extracts.

### Identification and quantification of major lutein esters by HPLC-MS

**Figure 4:**
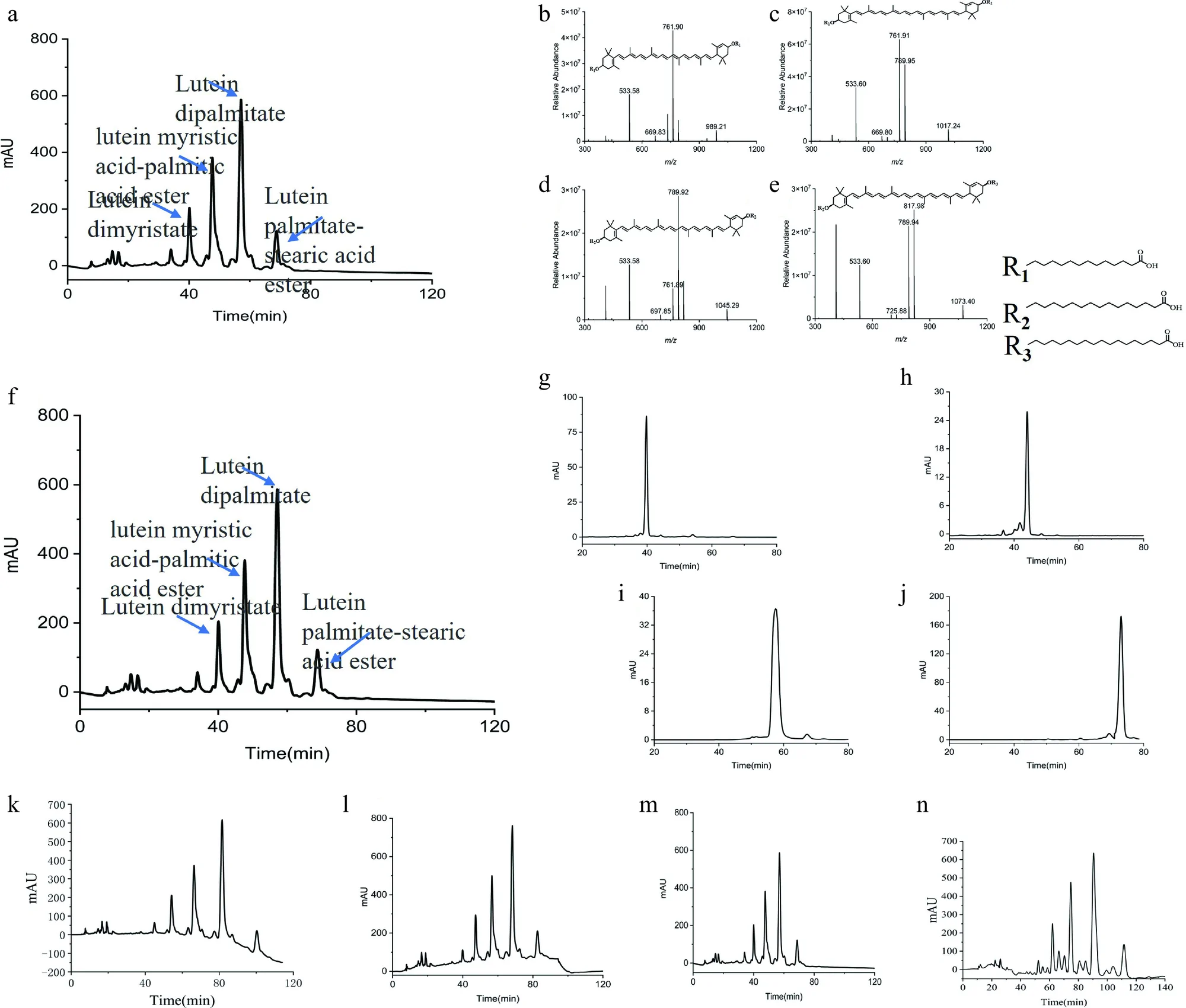
HPLC and HPLC-MS characterization of lutein esters and evaluation of chromatographic conditions. (a) HPLC chromatogram of marigold extract. (b) HPLC-MS ion fragment spectrum of lutein dimyristate. (c) HPLC-MS ion fragment spectrum of lutein myristic acid–palmitic acid ester. (d) HPLC-MS ion fragment spectrum of lutein dipalmitate. (e) HPLC-MS ion fragment spectrum of lutein palmitate–stearic acid ester. (f) HPLC chromatogram of marigold extract. (g) HPLC chromatogram of lutein dimyristate. (h) HPLC chromatogram of lutein myristic acid–palmitic acid ester. (i) HPLC chromatogram of lutein dipalmitate. (j) HPLC chromatogram of lutein palmitate–stearic acid ester. (k)–(m) HPLC chromatograms obtained using different mobile phases: (k) mobile phase A, (l) mobile phase B, and (m) mobile phase C. (n) Detection chromatogram obtained using the C30 chromatographic column.

The marigold extract was analyzed using a triple quadrupole HPLC-MS system. The retention times and HPLC-MS conditions were first determined according to Section 'HPLC-MS analysis'. The lutein esters were identified by comparing their fragment ion information with reference data. Multiple sets of unique fragment ions were used to confirm the structure of each ester. LC-MS analysis revealed the presence of four predominant lutein ester species in the extract, including lutein dimyristate, lutein myristate-palmitate, lutein dipalmitate, and lutein palmitate-stearate (Supplementary Table S1). The HPLC chromatogram of these four lutein esters is shown in Fig. 4a, and their corresponding MS ion fragment spectra are presented in Fig. 4b–e, respectively. Using four lutein ester reference compounds, standard curves were established for quantitative analysis, as summarized in Supplementary Table S2. The corresponding HPLC chromatograms are shown in Fig. 4f–j, representing lutein dimyristate, lutein myristate-palmitate, lutein dipalmitate, and lutein palmitate-stearate, respectively.

Due to the complex composition of the non-saponified marigold extract, optimizing separation conditions was critical. A combination of acetonitrile, ethyl acetate, and methanol was employed as the mobile phase. Three different mobile phase gradients were tested: A (ethyl acetate:acetonitrile:methanol = 56:24:20, v/v/v, Fig. 4k); B (52.5:22.5:25, v/v/v, Fig. 4l); and C (49:21:30, v/v/v, Fig. 4m). Mobile phase C provided stable baselines and good resolution for the four lutein esters and was therefore selected as the final condition. Both C18 and C30 chromatographic columns were evaluated. C18 provided good separation of unsaponifiable carotenoids, whereas C30 was mainly suitable for lutein esters. The C18 column was ultimately chosen for its short separation time and cost-effectiveness (Fig. 4m, n).

### Evaluation of 'conversion factor T'

The 'conversion factor T' method was applied to quantitatively assess the major lutein esters in marigold extracts. To establish the conversion factors, 1.0, 2.5, 5.0, 10.0, 20.0, and 30.0 μL of the test solutions were accurately injected and analyzed by HPLC. Using the formula described in Section 'Sample preparation' (Eq. [3]), the conversion factors T*_d_*, T_*m*_, and T_*p*_ of lutein dipalmitate to lutein dimyristate, lutein myristate-palmitate, and lutein palmitate-stearate were calculated. The obtained values were 0.903, 0.964, and 1.035, respectively, with RSDs less than 1%, indicating excellent stability and minimal influence of injection volume (Supplementary Table S3).

The robustness of the 'conversion factor T' method was further evaluated under different instrumental conditions. Two chromatographic columns, Bonnasll AQ C18 and Welchrom XB C18, were tested on Qinfang LC-3020 and Panna Brave HPLC systems. The RSD and RCF values obtained were all below 2%, demonstrating that different instruments and columns had negligible impact. The method was also applied to lutein ester solutions at various concentrations (6.25, 8.5, 10, 12.5, and 50 μg/mL). Concentrations below 12.5 μg/mL showed RSDs > 4%, whereas values above 12.5 μg/mL exhibited good reproducibility. The influence of column temperature (25, 30, 35, 40 °C) and flow rate (0.1, 0.2, 0.3, 0.4 mL/min) was also tested; RSD and RCF remained below 2% (Supplementary Table S4), confirming the method’s robustness.

### Determination of lutein content based on MIP/Ni_3_(HITP)_2_-MOF electrochemical sensing and conversion factor T

Conventional determination of lutein content generally requires saponification to convert lutein esters into free lutein prior to analysis. In contrast, the proposed strategy combines electrochemical determination of lutein dipalmitate with the conversion factor T method to estimate the contents of the remaining major lutein esters and subsequently calculate the total lutein content.

**Figure 5:**
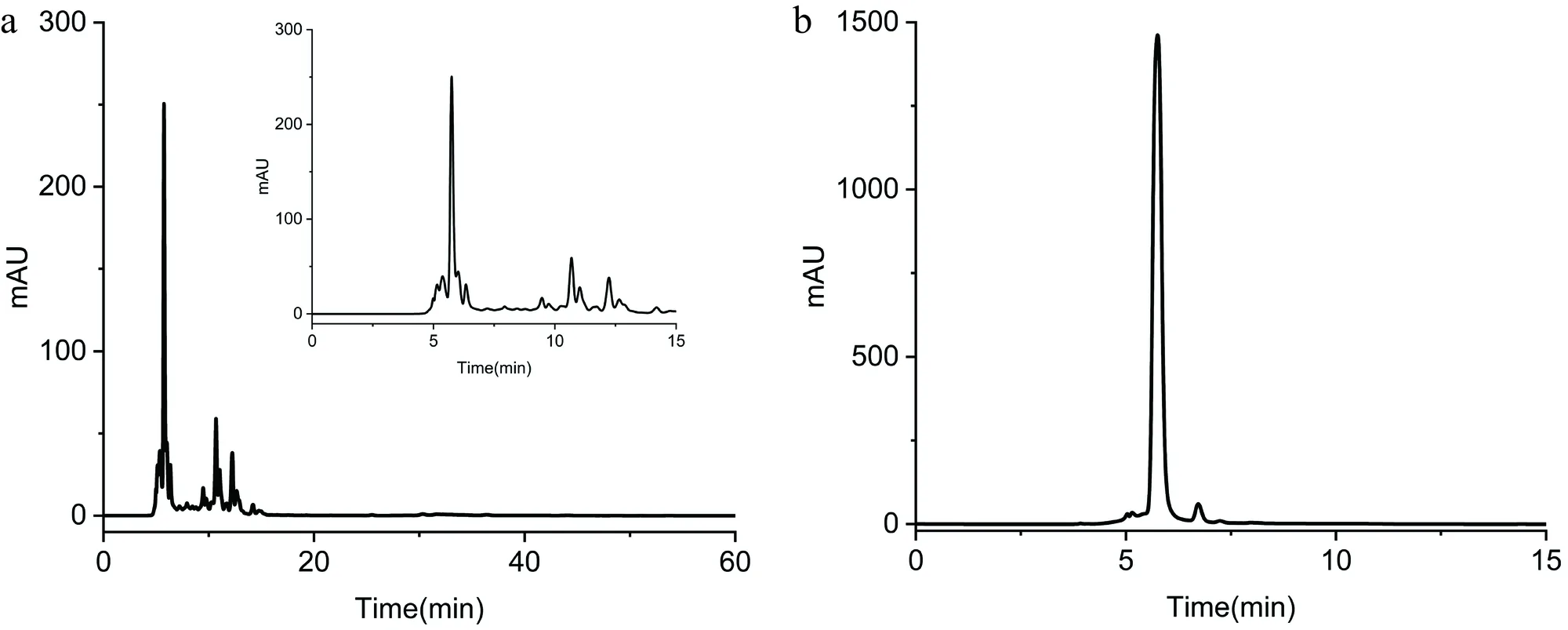
HPLC chromatograms of free lutein detection. (a) Saponification sample; the inset shows an enlarged view of the chromatogram from 0 to 15 min. (b) Lutein reference standard.

After saponification, the lutein content determined by the conventional method was 71.73 mg/g (Fig. 5). Using the proposed method, lutein dipalmitate was first quantified by the MIP/Ni_3_(HITP)_2_-MOF/SPCE sensor, and the contents of lutein dimyristate, lutein myristate-palmitate, and lutein palmitate-stearate were subsequently calculated using their corresponding conversion factors. Based on the total ester content, the lutein content was calculated to be 62.63 mg/g (Supplementary Table S5).

The difference between the two methods was only 8.80%, indicating good agreement between the proposed strategy and the conventional saponification method. The slightly lower value obtained by the proposed method may be attributed to the presence of minor lutein ester species that were not included in the conversion model. In addition, a small amount of free lutein was detected in the sample, with a calculated content of approximately 2.79 mg/g. These results demonstrate that the combination of electrochemical sensing and the conversion factor T method can provide a rapid and reliable alternative for lutein determination without the need for saponification.

### Application of electrochemical sensing and conversion factor T method to commercial eye-care products

Commercial eye-care products were analyzed to further evaluate the practical applicability of the proposed electrochemical sensing–conversion factor T strategy. The lutein content determined by the conventional saponification method was 3.72 mg/g, whereas the value obtained using the proposed method was 3.60 mg/g (Supplementary Table S6), demonstrating excellent agreement between the two methods.

The slightly lower value obtained by the proposed method may be attributed to minor lutein ester species that were not included in the conversion model. Nevertheless, the results indicate that the electrochemical sensing–conversion factor T strategy can accurately determine lutein content in commercial products while avoiding the time-consuming saponification process. Therefore, the proposed method shows considerable potential for routine quality control of marigold extracts and lutein-containing health products.

## Discussion

The stability of the conversion factor (T) is essential for the reliability of the proposed quantitative strategy. In this study, the T values remained highly consistent under different chromatographic systems, indicating that the established conversion factors possess good robustness. This stability may be associated with the structural similarity among the four major lutein esters. These compounds share the same lutein backbone and differ primarily in the esterified fatty acid chains. Therefore, under identical analytical conditions, their physicochemical properties and detector responses are expected to vary in a similar manner, contributing to relatively stable response relationships. Although the exact mechanism underlying the stability of the conversion factors requires further investigation, the high structural similarity among these lutein esters provides a reasonable explanation for the experimentally observed robustness of the T values.

The excellent analytical performance of the proposed sensor can be attributed to the synergistic effect of the Ni_3_(HITP)_2_-MOF and the molecularly imprinted polymer. Owing to its high electrical conductivity, large specific surface area, and abundant electroactive sites, the Ni_3_(HITP)_2_-MOF facilitates electron transfer and enhances the electrochemical response toward lutein dipalmitate. Meanwhile, the molecularly imprinted layer provides specific recognition cavities that match the target molecule in terms of size, shape, and functional groups, thereby improving the selectivity of the sensor and reducing interference from structurally related compounds. Compared with conventional electrochemical sensors without molecular imprinting, the proposed MIP-based sensor provides enhanced molecular recognition capability and improved selectivity toward lutein dipalmitate. The integration of specific imprinting sites with the conductive Ni_3_(HITP)_2_-MOF substrate enables effective discrimination of the target molecule from structurally related compounds, which is particularly advantageous for the analysis of complex natural product matrices. By integrating the electrochemical sensor with the conversion factor (T) strategy, the contents of the other three major lutein esters can be rapidly estimated from the electrochemically determined lutein dipalmitate concentration, eliminating the need for multiple reference standards and significantly improving analytical efficiency.

Compared with conventional chromatographic methods, the proposed strategy provides several practical advantages. The electrochemical sensing process is simple, rapid, and does not require saponification, thereby reducing sample pretreatment time and minimizing the potential degradation of lutein esters during alkaline hydrolysis. In addition, only one target analyte needs to be directly quantified, while the contents of the remaining major lutein esters can be calculated using the established conversion factors, which simplifies routine analysis and reduces the consumption of reference standards. Moreover, owing to its rapid response, simplified operation, and reduced dependence on multiple reference standards, the proposed strategy shows great potential for routine quality control and rapid screening of lutein ester compositions in marigold extracts and related commercial products. With further validation using a wider range of samples, this method may provide a practical analytical tool for industrial quality assessment and large-scale monitoring of lutein-containing products. Nevertheless, the present conversion factor model was established based on the four predominant lutein esters commonly found in marigold. Therefore, its applicability to samples containing substantially different lutein ester compositions or relatively high proportions of minor lutein esters should be further evaluated in future studies.

## Conclusion

The main purpose of this work was to develop a rapid and reliable strategy for the quantitative analysis of major lutein esters in marigold extracts. A conversion factor (T) strategy was established based on the proportional relationship among the four major lutein esters, using lutein dipalmitate as a representative reference compound. Furthermore, a molecularly imprinted electrochemical sensor based on Ni_3_(HITP)_2_-MOF was constructed for the selective and sensitive detection of lutein dipalmitate.

By integrating the molecular recognition capability of the MIP-based electrochemical sensor with the quantitative correlation provided by the conversion factor strategy, multiple lutein esters can be evaluated through the determination of a single target analyte. Compared with conventional analytical approaches, the proposed method avoids complicated saponification procedures, reduces the dependence on multiple reference standards, and provides a more efficient approach for lutein ester analysis.

Overall, the proposed electrochemical sensing-conversion strategy provides a rapid, convenient, and reliable platform for the quality evaluation of marigold extracts and lutein-containing products. The comparison of this method with existing quantitative approaches for lutein esters is summarized in Table 1, further demonstrating its advantages in terms of analytical efficiency, accuracy, and operational simplicity. With further validation using more diverse sample types, this strategy shows potential for application in routine quality control, rapid screening, and industrial monitoring of natural products.

**Table 1:** Comparison of existing methods and new methods for quantitative detection of lutein esters.

Number	Quantitative method	Merits/demerits	Ref.
1	Lutein equivalent calculation of lutein ester	Other substances with the same absorption value are covered, and the calculated value is high	[35]
2	Lutein standard curve calculation of lutein ester	The molecular weight difference is significant, and the detection is interfered by other substances, which is easy to cause large error in the calculated value	[36]
3	Lutein equivalent calculation of lutein ester	Other substances with the same absorption value are covered, and the calculated value is high	[37]
4	Saponified lutein ester, calculate lutein content	Saponification causes large loss of lutein	[38]
5	Only one major lutein ester content was calculated	Detection of material is too little; the error is too large	[39]
6	Saponified lutein ester, calculate lutein content	Saponification causes large loss of lutein	[40]
7	Only carotenoid esters were quantified; no specific lutein esters were quantified, and lutein content was quantified	The detection substance is not clear, and the calculation content error is large	[41]
8	MIP/Ni_3_(HITP)_2_-MOF electrochemical sensing-conversion factor T strategy	Direct detection of lutein esters, simultaneous and accurate quantification of a variety of major lutein esters, simplified operation, high detection efficiency	This work

## SUPPLEMENTARY DATA

Supplementary data to this article can be found online.

## Data Availability

The original contributions presented in the study are included in the article/Supplementary Material. Further inquiries can be directed to the corresponding author.
