# Supplementary material for: A molecularly imprinted electrochemical sensor integrated with conversion factor T strategy for rapid and selective determination of lutein esters in marigold extracts
Source: Eng Life Sci. 2026 Aug 24;26(8):e010. doi: 10.48130/els-0026-0010 (PMC13599645; doi:10.48130/els-0026-0010)
Supplement: Supplementary file 1 [file els-2026-26-8-0010-S1.zip › 10.48130_els-0026-0010-Suppl-TableS2.pdf]

Table S2 The regression equations, limits of detections (LODs), and limits of quantity (LOQs) of eleven compounds.

Analytes	Regression equation	Linear range (ug/mL)	R	LOD (ug/mL)	LOQ (ug/mL)
lutein	y = 11.394x + 37.369	12.50-330	0.9993	0.045	0.148
Lutein dimyristate	y = 8.9731x - 9.2229	19.23-500	0.9990	0.032	0.104
Lutein myristate -palmitate	y = 9.3321x - 20.499	19.23-500	0.9993	0.053	0.176
Lutein dipalmitate	y = 9.2704x + 16.55	19.23-500	0.9992	0.056	0.181
Lutein palmitate-stearate	y = 9.8217x - 34.68	19.23-500	0.9993	0.038	0.129
