# Supplementary material for: A molecularly imprinted electrochemical sensor integrated with conversion factor T strategy for rapid and selective determination of lutein esters in marigold extracts
Source: Eng Life Sci. 2026 Aug 24;26(8):e010. doi: 10.48130/els-0026-0010 (PMC13599645; doi:10.48130/els-0026-0010)
Supplement: Supplementary file 1 [file els-2026-26-8-0010-S1.zip › 10.48130_els-0026-0010-Suppl-TableS3.pdf]

**Table S3** Conversion factor T assay results for different lutein esters (n=3)

| Injection volume (μL) | Conversion factor T | | |
|---|---|---|---|
| | Td | Tm | Tp |
| 1.0 | 0.899 | 0.958 | 1.037 |
| 2.5 | 0.907 | 0.962 | 1.033 |
| 5 | 0.901 | 0.969 | 1.039 |
| 10 | 0.904 | 0.966 | 1.036 |
| 20 | 0.905 | 0.964 | 1.031 |
| 30 | 0.907 | 0.962 | 1.034 |
| average value | 0.903 | 0.964 | 1.035 |
| RSD per cent | 0.362 | 0.392 | 0.280 |
