# Supplementary material for: A molecularly imprinted electrochemical sensor integrated with conversion factor T strategy for rapid and selective determination of lutein esters in marigold extracts
Source: Eng Life Sci. 2026 Aug 24;26(8):e010. doi: 10.48130/els-0026-0010 (PMC13599645; doi:10.48130/els-0026-0010)
Supplement: Supplementary file 1 [file els-2026-26-8-0010-S1.zip › 10.48130_els-0026-0010-Suppl-TableS4.pdf]

Table S4 Effects of different instruments, columns, column temperatures, and flow rates on RSD and RCF (n = 3)

		**Td**	**Tm**	**Tp**
Qinfang LC-3020	Bonnasll AQ C18	0.904	0.966	1.036
	Welchrom XB C18	0.907	0.969	1.038
Panna Brave	Bonnasll AQ C18	0.894	0.956	1.021
	Welchrom XB C18	0.887	0.951	1.019
	Mean	0.898	0.9605	1.029
	RSD (%)	1.03	0.88	0.96
	RCF(%)	1.23	0.78	1.01
Flow rates (mL/min)	0.1	0.891	0.948	1.022
	0.2	0.902	0.963	1.033
	0.3	0.908	0.969	1.041
	0.4	0.904	0.966	1.036
	Mean	0.901	0.962	1.033
	RSD (%)	0.81	0.97	0.78
	RCF(%)	1.32	0.91	1.25
Column Temperatures (°C)	25	0.889	0.951	1.024
	30	0.904	0.966	1.036
	35	0.908	0.971	1.043
	40	0.913	0.974	1.048
	Mean	0.903	0.966	1.038
	RSD (%)	1.14	1.06	1.00
	RCF(%)	1.39	1.23	1.16
