# Supplementary material for: A molecularly imprinted electrochemical sensor integrated with conversion factor T strategy for rapid and selective determination of lutein esters in marigold extracts
Source: Eng Life Sci. 2026 Aug 24;26(8):e010. doi: 10.48130/els-0026-0010 (PMC13599645; doi:10.48130/els-0026-0010)
Supplement: Supplementary file 1 [file els-2026-26-8-0010-S1.zip › 10.48130_els-0026-0010-Suppl-TableS5.pdf]

Table S5 Comparison of lutein contents determined by conventional saponification and electrochemical sensing–conversion factor T strategy (n = 3)

Section	Saponified lutein (mg/g)	Electrochemically determined lutein dipalmitate (mg/g)	Lutein dimyristate (mg/g)	Lutein myristate-palmitate (mg/g)	Lutein palmitate-stearate (mg/g)	Calculated lutein content (mg/g)
1	73.84	48.35	18.04	35.32	12.17	62.86
2	71.60	48.06	18.43	34.99	11.92	62.61
3	69.74	47.81	18.18	34.78	12.38	62.42
Mean ± SD	71.73 ± 2.06	48.07 ± 0.27	18.22 ± 0.20	35.03 ± 0.27	12.16 ± 0.23	62.63 ± 0.22
