# Supplementary material for: A molecularly imprinted electrochemical sensor integrated with conversion factor T strategy for rapid and selective determination of lutein esters in marigold extracts
Source: Eng Life Sci. 2026 Aug 24;26(8):e010. doi: 10.48130/els-0026-0010 (PMC13599645; doi:10.48130/els-0026-0010)
Supplement: Supplementary file 1 [file els-2026-26-8-0010-S1.zip › 10.48130_els-0026-0010-Suppl-TableS6.pdf]

Table S6 Application of the electrochemical sensing–conversion factor T strategy to commercial eye-care products (n = 3)

**Section**	**Saponified lutein (mg/g)**	**Electrochemically determined lutein dipalmitate (mg/g)**	**Lutein dimyristate (mg/g)**	**Lutein myristate-palmitate (mg/g)**	**Lutein palmitate-stearate (mg/g)**	**Calculated lutein content (mg/g)**
1	3.72	2.78	0.98	1.97	0.72	3.55
2	3.79	2.83	1.00	2.01	0.73	3.63
3	3.65	2.82	0.99	2.00	0.73	3.61
Mean ± SD	3.72 ± 0.07	2.81 ± 0.03	0.99 ± 0.01	2.00 ± 0.02	0.73 ± 0.01	3.60 ± 0.04
