# Supplementary material for: A molecularly imprinted electrochemical sensor integrated with conversion factor T strategy for rapid and selective determination of lutein esters in marigold extracts
Source: Eng Life Sci. 2026 Aug 24;26(8):e010. doi: 10.48130/els-0026-0010 (PMC13599645; doi:10.48130/els-0026-0010)
Supplement: Supplementary file 1 [file els-2026-26-8-0010-S1.zip › 10.48130_els-0026-0010-Suppl-TableS1.pdf]

Table S1 Mass spectrometry ion peaks and fragment data of four lutein esters in marigold extract

Name	Characteristic ion fragments
Lutein dimyristate	989.21 [M]+, 761.90 [M+H-228 (C14)]+, 669.83 [M+H-228 (C14)-92], 533.58 [M+H-228 (C+14)-228 (C14)]+
Lutein myristate-palmitate	1017.24 [M]+, 789.95 [M+H-228 (C14)]+, 761.91 [M+H-256 (C16)]+, 669.80 [M+H-256 (C16)-92], 533.60 [M+H-228 (C+14)-256 (C16)]+
Lutein dipalmitate	1045.29 [M]+, 789.92 [M+H-256 (C16)]+, 697.85 [M+H-256 (C16)-92], 533.58 [M+H-256 (C+16)-256 (C16)]+
Lutein palmitate-stearate	1073.40 [M]+, 817.98 [M+H-[M+H-256 (C16)]+, 789.94 [M+H-284 (C18)]+, 725.88 [M+H-256 (C16)-92], 533.60 [M+H-228 (C+14)-256 (C16)]+
